# Pericardial Agenesis: The Significance of Multimodality Imaging in Diagnosis

**DOI:** 10.7759/cureus.82718

**Published:** 2025-04-21

**Authors:** Mehak Gupta, Tom Butler, Anikethana Appaji, Chun Shing Kwok

**Affiliations:** 1 Cardiology, University Hospitals of North Midlands NHS Foundation Trust, Stoke on Trent, GBR; 2 Medicine, University of Southampton, Southampton, GBR; 3 Cardiology, Mid Cheshire Hospitals NHS Foundation Trust, Crewe, GBR

**Keywords:** cardiac magnetic resonance imaging, computed tomography, incidental findings, multimodality imaging, pericardial agenesis

## Abstract

Pericardial agenesis is a rare pericardial defect that can be complete or partial. Often an incidental finding, most patients are asymptomatic at time of detection but some can present with symptoms of atypical chest pain, dyspnoea, palpitations or dizziness. There can be a delay in diagnosis as imaging is essential to confirm the diagnosis although there may be subtle signs on radiographic imaging and electrocardiogram. Both cardiac magnetic resonance imaging and computed tomography can be used to diagnose the condition and distinguish between complete and partial pericardial agenesis, although cardiac magnetic resonance has been suggested as the investigation of choice. Recognition of the condition is important as it poses a risk of complications, particularly in those with partial pericardial agenesis. Most asymptomatic patients with complete pericardial agenesis are managed conservatively. In this case report, we describe a case of a 45-year-old woman who presented with pleuritic chest pain and haemoptysis who was found to have complete pericardial agenesis on workup.

## Introduction

Pericardial agenesis (PA) is a rare pericardial defect that arises due to abnormal embryonal development [1]. The prevalence (inclusive of cases with other cardiopulmonary anomalies) is approximately 0.002-0.004% according to a surgical and pathological series [2]. PA can be classified into two forms: complete and partial. Complete form is the most frequent. It can be left-sided (70%), right-sided (17%), or bilateral (9%) [3]. The wide spectrum of the clinic presentation ranges from asymptomatic cases to fatal complications [3].

Most patients are asymptomatic at the time of detection but some can present with symptoms of atypical chest pain, dyspnoea, palpitations or dizziness [4]. There can be a delay in diagnosis as imaging is essential to confirm the diagnosis, although there may be subtle signs on radiographic imaging and electrocardiogram. Both cardiac magnetic resonance imaging and computed tomography can be used to diagnose the condition and distinguish between complete and partial pericardial agenesis, although cardiac magnetic resonance has been suggested as the investigation of choice [3,5]. Recognition of the condition is important as it poses a risk of complications, particularly those with partial pericardial agenesis. Most asymptomatic patients with complete PA are managed conservatively [6]. In some cases it does not impact clinical care of PA, and has a reduced clinical relevance, and it is detected as an incidental finding in imaging studies or during surgery or posthumously [3,7].

In this manuscript, we present a case of a patient who was incidentally found to have a congenital absence of the pericardium.

## Case presentation

**Figure 1:**
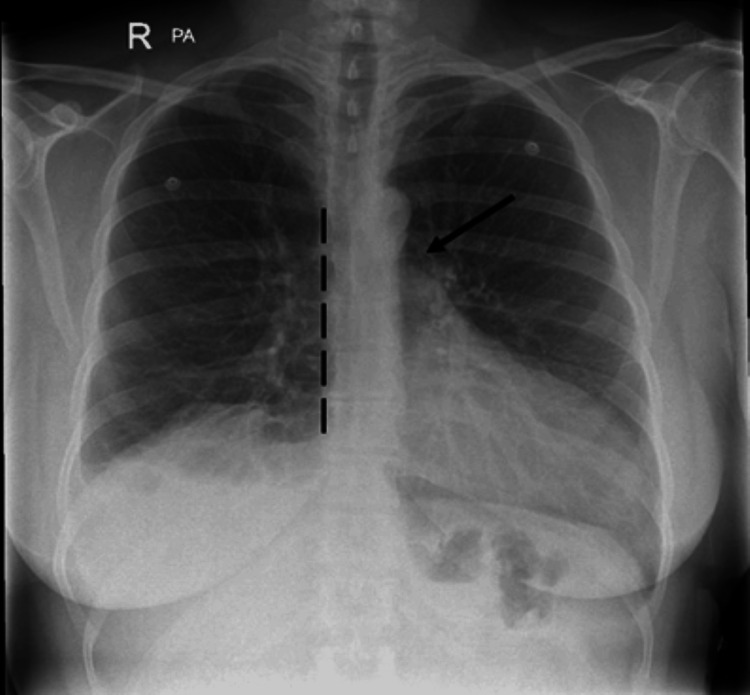
There is leftward displacement of cardiac silhouette with flattening of left heart border (dotted black line). Lucent area between aorta and pulmonary artery (black arrow).

A 45-year-old female presented to the hospital with pleuritic chest pain for one week and haemoptysis. This was right-sided and radiated to the back and got progressively worse. She also had shortness of breath and was believed to have a recent COVID-19 infection. Her past medical history includes asthma, keratoconjuctivitis, and piriformis syndrome. She started hormone replacement therapy about five months ago as she had been perimenopausal for over a year. Prior to admission she was active, going to the gym daily. She had a family history of deep vein thrombosis in her father and grandmother. Her medications included montelukast 10 mg daily, lansoprazole 15 mg daily, Ellest Duet 1 mg daily, DuoResp Spiromax inhaler, and Ventolin inhaler. On examination, her blood pressure was 154/86 mmHg, respiratory rate was 17 breaths per minute, heart rate was 99 beats per minute, temperature was 36.4°C, and saturations were 96% on room air. Other physical examination findings were unremarkable. Her blood tests showed a D-dimer of 1727 nanogram/milliliter (normal <500 nanogram/milliliter). Her electrocardiogram showed a sinus rhythm of 96 beats per minute with T wave inversion in the inferior and precordial leads. Her chest X-ray is shown in Figure 1.

**Figure 2:**
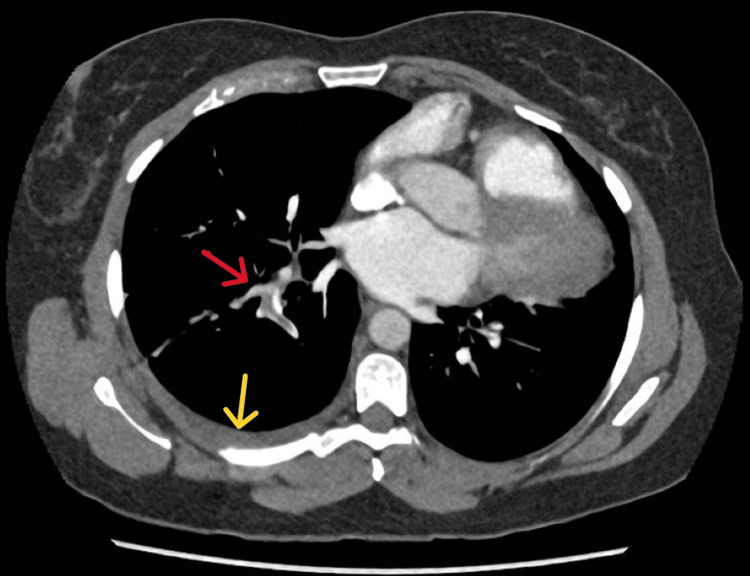
There is a blood clot in the segmental branches of the right pulmonary artery (red arrow) and small right pleural effusion (yellow arrow).

A diagnosis of pulmonary embolism was initially suspected and a computerised tomography pulmonary angiogram (CTPA) was requested. This showed a pulmonary embolism involving the segmental branches of the right lower lobe and a small right-sided pleural effusion (Figure 2). An incidental finding of excessive levoposition of the heart with absence of pericardium was also seen. She was started on anticoagulation and discharged.

She was thereafter reviewed in the cardiology clinic. At the time of review, she reported no cardiovascular symptoms. Her cardiovascular examination was unremarkable except an elevated blood pressure was noted. A new diagnosis of hypertension was made and she was initiated on ramipril. Thereafter, she underwent an echocardiogram which was reported as showing very unconventional cardiac orientation with grossly oblique views. There was an overall normal left ventricular function. The right heart was reported as mildly dilated with mild impairment.

**Figure 3:**
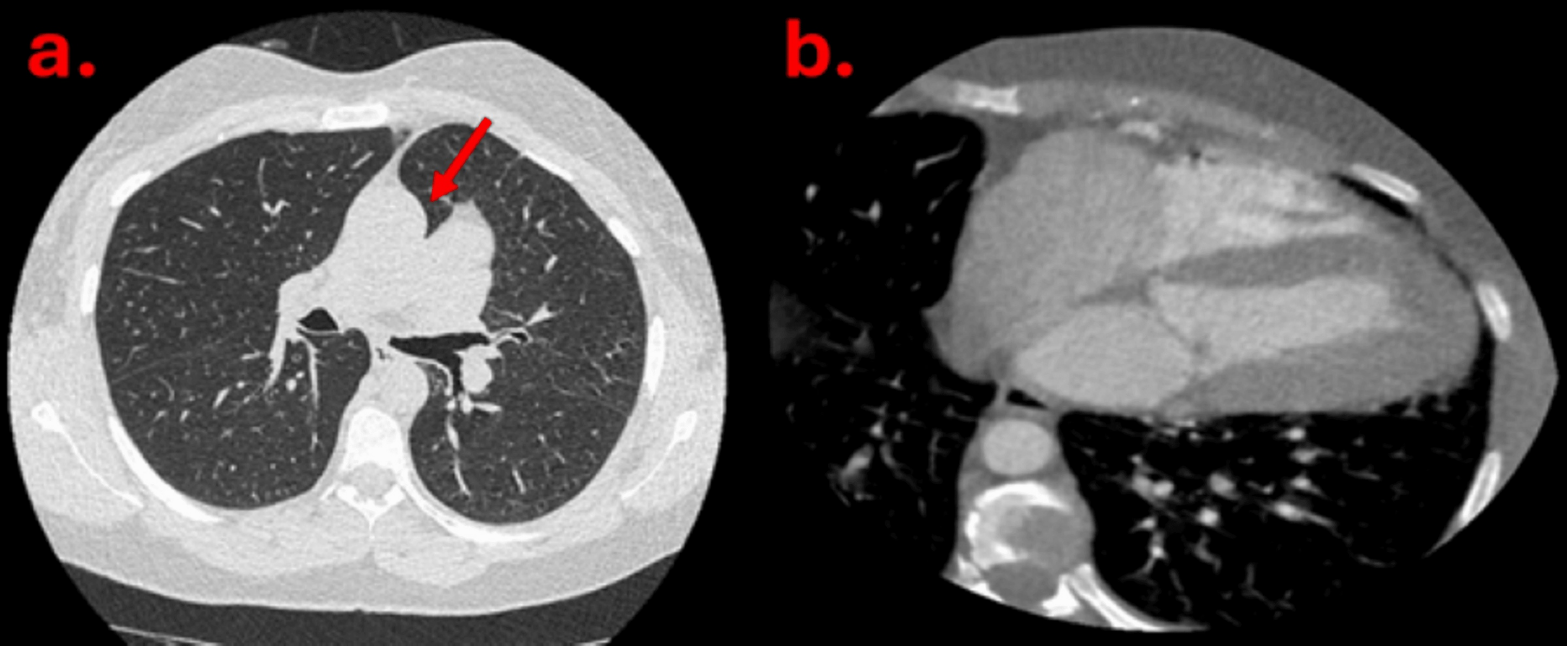
There is interposition of the lung tissue between the aorta and pulmonary artery (solid red arrow in a). The heart is positioned in the left hemithorax with absent pericardium (b).

Cardiac CT was carried out, which demonstrated normal origin of coronary arteries with no significant coronary artery disease. Additional findings are as shown in Figure 3A, 3B.

**Figure 4:**
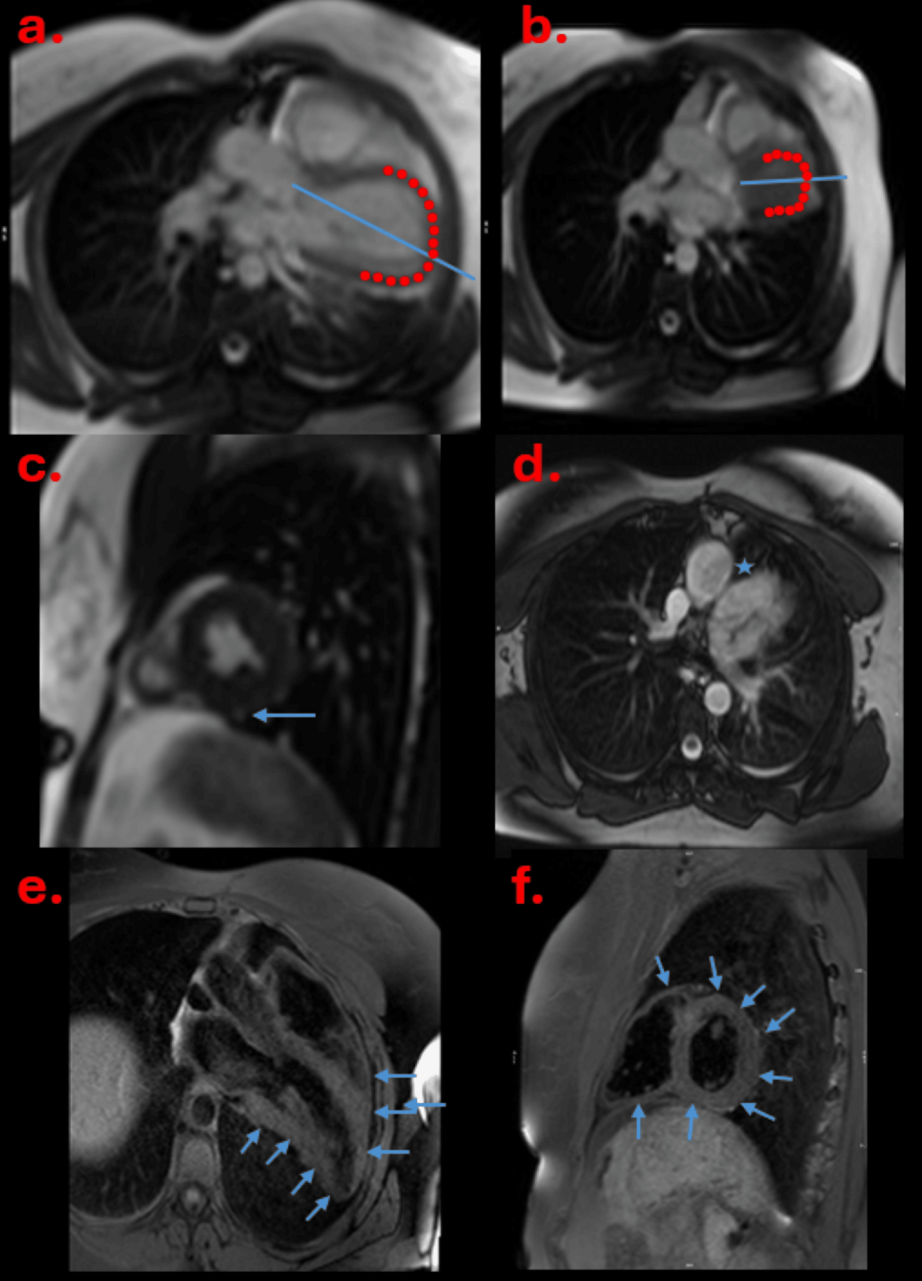
a and b show exaggerated cardiac motion in diastole (a) and systole (b). There is interposition of lung tissue between diaphragm and heart (c, solid blue arrow) whilst d shows interposition of lung tissue between aorta and pulmonary artery (solid blue star). e and f are the fast spin echo images demonstrating absent pericardium (solid blue arrows).

The right heart impairment prompted further evaluation with cardiac magnetic resonance imaging (MRI) to look at cardiac anatomy in greater detail and assess for shunts. Cardiac MRI demonstrated a normal biventricular size and function. The right ventricular volume was larger than the left ventricle but within normal limits. No significant shunts were detected and there was no scarring. The displacement of the heart into the left hemithorax with accentuated movement within the hemithorax on free breathing was noted (Figure 4A, 4B, quality degraded due to motion artefacts). There was interposition of lung tissue between the diaphragm and the heart (Figure 4C) and aorta and pulmonary artery (Figure 4D) which is a specific sign for pericardial agenesis. On T1 fast spin echo images, the pericardium was absent (Figure 4E, 4F).

Hypercoagulability screen and CT abdomen and pelvis were also requested which were all normal. She remains well in herself, is asymptomatic, and hence she is currently being followed up in clinic.

## Discussion

The pericardium is a sac surrounding the heart and great vessels which has the role to protect these structures against injury and infection and fix the heart to the mediastinum. It comprises of an outer part called fibrous pericardium and an inner layer called serous pericardium [8]. Acute cardiac dilatation and the mechanical interactions of the heart chambers is limited by the relatively inelastic nature of the pericardium [9]. The pericardium originates from one of the three intraembryonic coelomic cavities during the fourth week of embryogenesis. Congenital agenesis of the pericardium arises as a result of failure of fusion of the pericardial cavities [5,9]. Other congenital cardiac anomalies, including atrial septal defect, patent ductus arteriosus, bicuspid aortic valve or pulmonary malformations, may be associated with pericardial defects [9].

Pericardial agenesis is an uncommon defect that is frequently diagnosed late due to a lack of symptoms and clinical awareness. Even though patients are asymptomatic, a minority may experience atypical chest pain secondary to exaggerated cardiac motion [10]. Other clinical manifestations include dyspnoea, dizziness or palpitations [4]. Often, patients have normal physical examinations and are asymptomatic. They are frequently diagnosed incidentally whilst undergoing thoracic imaging for other reasons as in our patient who underwent a CT pulmonary angiogram during her acute presentation to the hospital. It is commonly observed in males three times more than in females [11].

The electrocardiogram is mostly normal in partial PA. Alternatively, in complete left PA, it frequently shows right axis deviation associated with incomplete right bundle branch block [12]. Moreover, due to hypermobility of the heart in the chest, electrical alternans of both P and QRS potentials have also been described [12]. Chest radiographic findings are usually subtle and may be reported as normal. However, the diagnosis can be suspected based on some characteristic features such as leftward cardiac silhouette displacement in posteroanterior views, posterior position of the cardiac shadow in lateral images, snoopy sign which is the elongation of the left ventricular contour, prominent pulmonary artery, radiolucency as a result of lung tissue between the base of the heart and the diaphragm or between the aortic knuckle and the main pulmonary artery, or an obscured right heart border by an overlying spine [5].

In an evaluation from the Mayo Clinic, 10 patients with PA underwent echocardiography between 1982 and 1992. The features in declining order of frequency are: unusual acoustic windows, increased cardiac mobility, abnormal ventricular septal motion, and abnormal swinging motion of the heart [13]. The leftward shift of the heart from parasternal windows results in the imaging of a greater part of the right ventricle, giving the impression of a dilated right ventricle [13]. This often leads to the suspicion of cardiac shunts or, in some instances, arrhythmogenic cardiomyopathy. This was the case in our patient, which prompted further investigations.

Cardiac CT shows the absence of pericardium with levoposition of the heart. It is also useful for detecting the lung tissue between the main pulmonary artery and the aorta [14]. Cardiac magnetic resonance is the gold standard of diagnosis as it can display cine images. It can show absence of pericardium, abnormal site of the heart in the hemithorax with excessive apical motion and interposition of lung tissue, especially between the aorta and the pulmonary artery, which is considered a specific sign. Both can be used to assess the extent of the defect (complete or partial), the presence of herniated structures or other abnormalities [15].

There is no evidence to suggest that asymptomatic patients with complete PA require any treatment [6]. Alternatively, surgery is indicated in patients with symptomatic or complicated partial defects (ischaemic disease, cardiac chambers herniation). This could be patch closure, total pericardiectomy or enlargement of the defect for incarceration prevention [6]. Prognosis is still not well established due to both the rarity of the disease and extreme variability of clinical presentation and hence is an area for future research [16]. It is further unknown whether patients should require regular follow-up.

## Conclusions

Pericardial agenesis is a rare cardiac disorder which is frequently diagnosed incidentally due to lack of symptoms. Multimodality imaging such as echocardiogram, cardiac CT and cardiac MRI plays a major role in its diagnosis though cardiac MRI remains the gold standard investigation of choice. The majority of patients are asymptomatic and do not require any intervention.
